# 5-Bromo-2-methyl-3-(4-methyl­phenyl­sulfin­yl)-1-benzofuran

**DOI:** 10.1107/S160053681201389X

**Published:** 2012-04-04

**Authors:** Hong Dae Choi, Pil Ja Seo, Uk Lee

**Affiliations:** a Department of Chemistry, Dongeui University, San 24 Kaya-dong Busanjin-gu, Busan 614-714, Republic of Korea; b Department of Chemistry, Pukyong National University, 599-1 Daeyeon 3-dong, Nam-gu, Busan 608-737, Republic of Korea

## Abstract

In the title compound, C_16_H_13_BrO_2_S, the 4-methyl­phenyl ring makes a dihedral angle of 87.83 (6)° with the mean plane [mean deviation = 0.007 (1) Å] of the benzofuran fragment. In the crystal, mol­ecules are linked by weak C—H⋯O hydrogen bonds and Br⋯O contacts [3.099 (2) Å]. The crystal structure also exhibits π–π inter­actions between the furan and benzene rings of neighbouring mol­ecules [centroid–centroid distance = 3.637 (2) Å, inter­planar distance = 3.317 (2) Å and slippage = 1.492 (2) Å].

## Related literature

For background information and the crystal structures of related compounds, see: Choi *et al.* (2010**a* ▶,b* ▶). For a review of halogen bonding, see: Politzer *et al.* (2007 ▶).
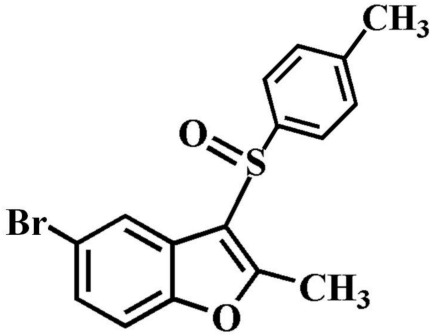


## Experimental

### 

#### Crystal data

C_16_H_13_BrO_2_S
*M* *_r_* = 349.23
Monoclinic, 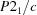

*a* = 14.3470 (2) Å
*b* = 11.2122 (1) Å
*c* = 9.6852 (1) Å
β = 107.556 (1)°
*V* = 1485.41 (3) Å^3^
*Z* = 4
Mo *K*α radiation
μ = 2.91 mm^−1^
*T* = 173 K
0.31 × 0.28 × 0.21 mm

#### Data collection

Bruker SMART APEXII CCD diffractometer
Absorption correction: multi-scan (*SADABS*; Bruker, 2009 ▶) *T*_min_ = 0.466, *T*_max_ = 0.579
14207 measured reflections
3671 independent reflections
2844 reflections with *I* > 2σ(*I*)
*R*_int_ = 0.032

#### Refinement

*R*[*F* ^2^ > 2σ(*F* ^2^)] = 0.034
*wR*(*F* ^2^) = 0.086
*S* = 1.03
3671 reflections
183 parameters
H-atom parameters constrained
Δρ_max_ = 0.55 e Å^−3^
Δρ_min_ = −0.77 e Å^−3^

### 

Data collection: *APEX2* (Bruker, 2009 ▶); cell refinement: *SAINT* (Bruker, 2009 ▶); data reduction: *SAINT*; program(s) used to solve structure: *SHELXS97* (Sheldrick, 2008 ▶); program(s) used to refine structure: *SHELXL97* (Sheldrick, 2008 ▶); molecular graphics: *ORTEP-3* (Farrugia, 1997 ▶) and *DIAMOND* (Brandenburg, 1998 ▶); software used to prepare material for publication: *SHELXL97*.

## Supplementary Material

Crystal structure: contains datablock(s) global, I. DOI: 10.1107/S160053681201389X/xu5502sup1.cif

Structure factors: contains datablock(s) I. DOI: 10.1107/S160053681201389X/xu5502Isup2.hkl

Supplementary material file. DOI: 10.1107/S160053681201389X/xu5502Isup3.cml

Additional supplementary materials:  crystallographic information; 3D view; checkCIF report

## Figures and Tables

**Table 1:** Hydrogen-bond geometry (Å, °)

*D*—H⋯*A*	*D*—H	H⋯*A*	*D*⋯*A*	*D*—H⋯*A*
C5—H5⋯O1^i^	0.95	2.49	3.420 (3)	165
C9—H9*C*⋯O2^ii^	0.98	2.48	3.294 (3)	141

Symmetry codes: (i) 
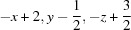
; (ii) 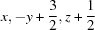.

**Fig. 1.**
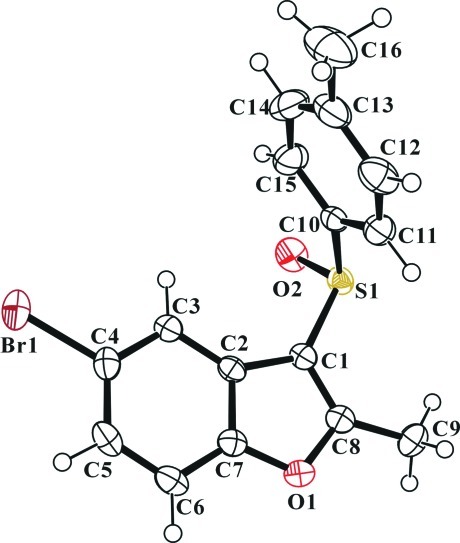
The molecular structure of the title compound with the atom numbering scheme. Displacement ellipsoids are drawn at the 50% probability level. H atoms are presented as small spheres of arbitrary radius.

**Fig. 2.**
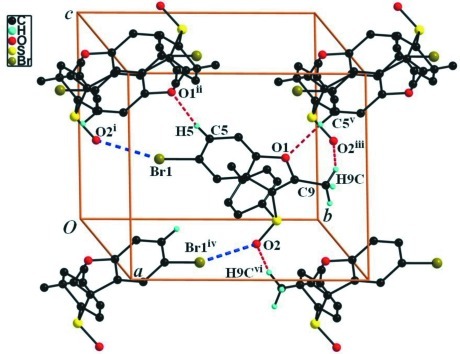
A view of the C—H···O and Br···O interactions (dotted lines) in the crystal structure of the title compound. H atoms non-participating in hydrogen-bonding were omitted for clarity. [Symmetry codes: (i) x,- y + 1/2,z + 1/2; (ii) - x + 2,y - 1/2,- z + 3/2; (iii) x,- y + 3/2 ,z + 1/2; (iv) x,- y + 1/2,z - 1/2.] (v) - x + 2,y + 1/2,- z + 3/2; (vi) x,- y + 3/2,z - 1/2;

**Fig. 3.**
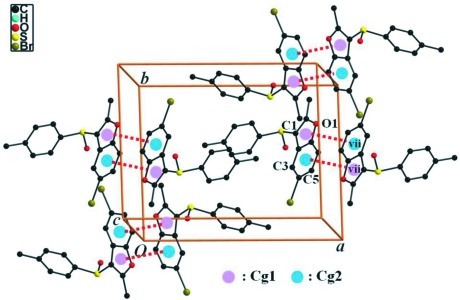
A view of the π···π interactions (dotted lines) in the crystal structure of the title compound. H atoms were omitted for clarity. [Symmetry codes: (vii) - x + 2,- y + 1,- z + 1.]

## supplementary crystallographic information

### Comment

As a part of our ongoing study of 5-bromo-2-methyl-1-benzofuran derivatives containing 3-(4-fluorophenylsulfinyl) (Choi *et al.*, 2010*a*) and 3-(4-chlorophenylsulfinyl) (Choi *et al.*, 2010*b*) substituents, we report herein the crystal structure of the title compound.

In the title molecule (Fig. 1), the benzofuran unit is essentially planar, with a mean deviation of 0.007 (1) Å from the least-squares plane defined by the nine constituent atoms. The dihedral angle between the 4-methylphenyl ring and the mean plane of the benzofuran fragment is 87.83 (6)°. In the crystal structure (Fig. 2), molecules are linked by weak C–H···O hydrogen bonds (Table 1) and Br···O halogen-bondings between the bromine atom and the O atom of the S=O unit [Br1···O2^i^ = 3.099 (2) Å, C4—Br1···O2^i^ = 163.52 (8)°] (Politzer *et al.*, 2007). The crystal packing (Fig. 3) also exhibits weak π···π interactions between the furan and benzene rings of neighbouring molecules, with a Cg1···Cg2^vii^ distance of 3.637 (2) Å and an interplanar distance of 3.317 (2) Å resulting in a slippage of 1.492 (2) Å (Cg1 and Cg2 are the centroids of the C1/C2/C7/O1/C8 furan ring and C2–C7 benzene ring, respectively).

### Experimental

3-Chloroperoxybenzoic acid (77%, 224 mg, 1.0 mmol) was added in small portions to a stirred solution of 5-bromo-2-methyl-3-(4-methylphenylsulfanyl)-1-benzofuran (300 mg, 0.9 mmol) in dichloromethane (30 mL) at 273 K. After being stirred at room temperature for 4 h, the mixture was washed with saturated sodium bicarbonate solution and the organic layer was separated, dried over magnesium sulfate, filtered and concentrated at reduced pressure. The residue was purified by column chromatography (hexane/ethyl acetate, 2:1 v/v) to afford the title compound as a colorless solid [yield 73%, m.p. 424–425 K; *R*_f_ = 0.45 (hexane/ethyl acetate, 2:1 v/v)]. Single crystals suitable for X-ray diffraction were prepared by slow evaporation of a solution of the title compound in ethyl acetate at room temperature.

### Refinement

All H atoms were positioned geometrically and refined using a riding model, with C—H = 0.95 Å for aryl and 0.98 Å for methyl H atoms. U_iso_(H) = 1.2*U*_eq_(C) for aryl and 1.5*U*_eq_(C) for methyl H atoms. The positions of methyl hydrogen atoms were optimized rotationally.

### Crystal data

**Table:**

C_16_H_13_BrO_2_S	*F*(000) = 704
*M**_r_* = 349.23	*D*_x_ = 1.562 Mg m^−^^3^
Monoclinic, *P*2_1_/*c*	Mo *K*α radiation, λ = 0.71073 Å
Hall symbol: -P 2ybc	Cell parameters from 5020 reflections
*a* = 14.3470 (2) Å	θ = 2.4–28.1°
*b* = 11.2122 (1) Å	µ = 2.91 mm^−^^1^
*c* = 9.6852 (1) Å	*T* = 173 K
β = 107.556 (1)°	Block, colourless
*V* = 1485.41 (3) Å^3^	0.31 × 0.28 × 0.21 mm
*Z* = 4	

### Data collection

**Table:**

Bruker SMART APEXII CCD diffractometer	3671 independent reflections
Radiation source: rotating anode	2844 reflections with *I* > 2σ(*I*)
Graphite multilayer monochromator	*R*_int_ = 0.032
Detector resolution: 10.0 pixels mm^-1^	θ_max_ = 28.3°, θ_min_ = 1.5°
φ and ω scans	*h* = −18→19
Absorption correction: multi-scan (*SADABS*; Bruker, 2009)	*k* = −14→14
*T*_min_ = 0.466, *T*_max_ = 0.579	*l* = −12→12
14207 measured reflections	

### Refinement

**Table:**

Refinement on *F*^2^	Primary atom site location: structure-invariant direct methods
Least-squares matrix: full	Secondary atom site location: difference Fourier map
*R*[*F*^2^ > 2σ(*F*^2^)] = 0.034	Hydrogen site location: difference Fourier map
*wR*(*F*^2^) = 0.086	H-atom parameters constrained
*S* = 1.03	*w* = 1/[σ^2^(*F*_o_^2^) + (0.0381*P*)^2^ + 0.8792*P*] where *P* = (*F*_o_^2^ + 2*F*_c_^2^)/3
3671 reflections	(Δ/σ)_max_ = 0.001
183 parameters	Δρ_max_ = 0.55 e Å^−^^3^
0 restraints	Δρ_min_ = −0.77 e Å^−^^3^

### Special details

**Table:**

Geometry. All esds (except the esd in the dihedral angle between two l.s. planes) are estimated using the full covariance matrix. The cell esds are taken into account individually in the estimation of esds in distances, angles and torsion angles; correlations between esds in cell parameters are only used when they are defined by crystal symmetry. An approximate (isotropic) treatment of cell esds is used for estimating esds involving l.s. planes.
Refinement. Refinement of F^2^ against ALL reflections. The weighted R-factor wR and goodness of fit S are based on F^2^, conventional R-factors R are based on F, with F set to zero for negative F^2^. The threshold expression of F^2^ > 2sigma(F^2^) is used only for calculating R-factors(gt) etc. and is not relevant to the choice of reflections for refinement. R-factors based on F^2^ are statistically about twice as large as those based on F, and R- factors based on ALL data will be even larger.

### Fractional atomic coordinates and isotropic or equivalent isotropic displacement parameters (Å^2^)

**Table:**

	*x*	*y*	*z*	*U*_iso_*/*U*_eq_	
Br1	0.79550 (2)	0.16838 (2)	0.52417 (3)	0.04775 (11)	
S1	0.73033 (4)	0.67240 (5)	0.20895 (5)	0.02775 (13)	
O1	0.94299 (11)	0.66889 (13)	0.57489 (16)	0.0296 (3)	
O2	0.73660 (12)	0.58439 (15)	0.09690 (16)	0.0386 (4)	
C1	0.81812 (15)	0.63651 (19)	0.3736 (2)	0.0253 (4)	
C2	0.83871 (14)	0.52469 (18)	0.4514 (2)	0.0244 (4)	
C3	0.80193 (15)	0.40884 (19)	0.4307 (2)	0.0282 (4)	
H3	0.7502	0.3872	0.3471	0.034*	
C4	0.84473 (17)	0.32647 (19)	0.5384 (2)	0.0312 (5)	
C5	0.92198 (17)	0.3549 (2)	0.6600 (2)	0.0339 (5)	
H5	0.9485	0.2953	0.7306	0.041*	
C6	0.96063 (16)	0.4685 (2)	0.6796 (2)	0.0320 (5)	
H6	1.0146	0.4889	0.7608	0.038*	
C7	0.91637 (15)	0.55073 (19)	0.5743 (2)	0.0258 (4)	
C8	0.88224 (15)	0.7186 (2)	0.4515 (2)	0.0267 (4)	
C9	0.89826 (18)	0.84651 (19)	0.4301 (3)	0.0356 (5)	
H9A	0.8605	0.8699	0.3316	0.053*	
H9B	0.9679	0.8607	0.4442	0.053*	
H9C	0.8770	0.8937	0.5002	0.053*	
C10	0.62293 (15)	0.63534 (19)	0.2582 (2)	0.0263 (4)	
C11	0.59828 (17)	0.7053 (2)	0.3594 (2)	0.0350 (5)	
H11	0.6393	0.7691	0.4059	0.042*	
C12	0.51270 (19)	0.6806 (2)	0.3919 (3)	0.0444 (6)	
H12	0.4960	0.7275	0.4627	0.053*	
C13	0.45106 (17)	0.5896 (2)	0.3241 (3)	0.0438 (6)	
C14	0.47699 (19)	0.5233 (3)	0.2218 (3)	0.0516 (7)	
H14	0.4349	0.4611	0.1728	0.062*	
C15	0.56315 (18)	0.5448 (2)	0.1883 (3)	0.0422 (6)	
H15	0.5802	0.4976	0.1181	0.051*	
C16	0.3583 (2)	0.5640 (3)	0.3611 (4)	0.0680 (9)	
H16A	0.3730	0.5576	0.4665	0.102*	
H16B	0.3299	0.4889	0.3157	0.102*	
H16C	0.3115	0.6290	0.3253	0.102*	

### Atomic displacement parameters (Å^2^)

**Table:**

	*U*^11^	*U*^22^	*U*^33^	*U*^12^	*U*^13^	*U*^23^
Br1	0.05663 (18)	0.02626 (14)	0.05713 (18)	0.00091 (11)	0.01230 (13)	0.00537 (11)
S1	0.0306 (3)	0.0274 (3)	0.0236 (2)	0.0031 (2)	0.0058 (2)	0.00431 (19)
O1	0.0262 (7)	0.0322 (8)	0.0285 (7)	−0.0028 (6)	0.0055 (6)	−0.0021 (6)
O2	0.0464 (10)	0.0442 (10)	0.0271 (8)	0.0076 (8)	0.0138 (7)	−0.0025 (7)
C1	0.0230 (10)	0.0281 (10)	0.0249 (10)	0.0010 (8)	0.0074 (8)	0.0031 (8)
C2	0.0211 (9)	0.0297 (10)	0.0231 (9)	0.0033 (8)	0.0077 (8)	0.0017 (8)
C3	0.0251 (10)	0.0289 (11)	0.0289 (10)	0.0016 (9)	0.0055 (8)	0.0001 (8)
C4	0.0342 (12)	0.0247 (10)	0.0364 (12)	0.0039 (9)	0.0130 (9)	0.0016 (9)
C5	0.0366 (12)	0.0357 (12)	0.0288 (11)	0.0144 (10)	0.0090 (9)	0.0058 (9)
C6	0.0286 (11)	0.0414 (13)	0.0239 (10)	0.0092 (10)	0.0048 (8)	−0.0005 (9)
C7	0.0230 (10)	0.0303 (11)	0.0254 (10)	0.0018 (8)	0.0092 (8)	−0.0019 (8)
C8	0.0243 (10)	0.0314 (11)	0.0267 (10)	0.0008 (9)	0.0108 (8)	0.0010 (8)
C9	0.0413 (13)	0.0290 (12)	0.0384 (12)	−0.0070 (10)	0.0152 (10)	−0.0013 (9)
C10	0.0237 (10)	0.0273 (10)	0.0241 (10)	0.0040 (8)	0.0015 (8)	0.0021 (8)
C11	0.0341 (12)	0.0352 (12)	0.0333 (12)	0.0022 (10)	0.0063 (10)	−0.0069 (9)
C12	0.0373 (13)	0.0571 (17)	0.0396 (14)	0.0115 (12)	0.0127 (11)	−0.0027 (12)
C13	0.0282 (12)	0.0504 (15)	0.0513 (15)	0.0074 (11)	0.0099 (11)	0.0130 (12)
C14	0.0347 (14)	0.0459 (16)	0.0705 (19)	−0.0131 (12)	0.0103 (13)	−0.0137 (14)
C15	0.0379 (13)	0.0411 (14)	0.0459 (14)	−0.0026 (11)	0.0099 (11)	−0.0151 (11)
C16	0.0352 (15)	0.089 (3)	0.082 (2)	0.0073 (16)	0.0223 (15)	0.025 (2)

### Geometric parameters (Å, º)

**Table:**

Br1—C4	1.898 (2)	C8—C9	1.477 (3)
Br1—O2^i^	3.0986 (16)	C9—H9A	0.9800
S1—O2	1.4895 (16)	C9—H9B	0.9800
S1—C1	1.755 (2)	C9—H9C	0.9800
S1—C10	1.794 (2)	C10—C15	1.369 (3)
O1—C8	1.368 (2)	C10—C11	1.382 (3)
O1—C7	1.378 (3)	C11—C12	1.384 (4)
C1—C8	1.357 (3)	C11—H11	0.9500
C1—C2	1.446 (3)	C12—C13	1.379 (4)
C2—C3	1.394 (3)	C12—H12	0.9500
C2—C7	1.394 (3)	C13—C14	1.377 (4)
C3—C4	1.390 (3)	C13—C16	1.507 (4)
C3—H3	0.9500	C14—C15	1.391 (4)
C4—C5	1.389 (3)	C14—H14	0.9500
C5—C6	1.379 (3)	C15—H15	0.9500
C5—H5	0.9500	C16—H16A	0.9800
C6—C7	1.379 (3)	C16—H16B	0.9800
C6—H6	0.9500	C16—H16C	0.9800
			
C4—Br1—O2^i^	163.52 (8)	C8—C9—H9B	109.5
O2—S1—C1	109.01 (10)	H9A—C9—H9B	109.5
O2—S1—C10	106.61 (10)	C8—C9—H9C	109.5
C1—S1—C10	98.26 (9)	H9A—C9—H9C	109.5
C8—O1—C7	106.57 (16)	H9B—C9—H9C	109.5
C8—C1—C2	107.50 (18)	C15—C10—C11	121.0 (2)
C8—C1—S1	121.98 (16)	C15—C10—S1	120.02 (17)
C2—C1—S1	130.52 (16)	C11—C10—S1	118.78 (17)
C3—C2—C7	119.24 (19)	C10—C11—C12	118.8 (2)
C3—C2—C1	136.39 (19)	C10—C11—H11	120.6
C7—C2—C1	104.36 (18)	C12—C11—H11	120.6
C4—C3—C2	116.68 (19)	C13—C12—C11	121.7 (2)
C4—C3—H3	121.7	C13—C12—H12	119.2
C2—C3—H3	121.7	C11—C12—H12	119.2
C5—C4—C3	122.9 (2)	C14—C13—C12	117.9 (2)
C5—C4—Br1	117.37 (17)	C14—C13—C16	121.2 (3)
C3—C4—Br1	119.72 (17)	C12—C13—C16	120.9 (3)
C6—C5—C4	120.9 (2)	C13—C14—C15	121.8 (2)
C6—C5—H5	119.6	C13—C14—H14	119.1
C4—C5—H5	119.6	C15—C14—H14	119.1
C7—C6—C5	116.1 (2)	C10—C15—C14	118.8 (2)
C7—C6—H6	122.0	C10—C15—H15	120.6
C5—C6—H6	122.0	C14—C15—H15	120.6
O1—C7—C6	125.05 (19)	C13—C16—H16A	109.5
O1—C7—C2	110.73 (18)	C13—C16—H16B	109.5
C6—C7—C2	124.2 (2)	H16A—C16—H16B	109.5
C1—C8—O1	110.84 (18)	C13—C16—H16C	109.5
C1—C8—C9	133.5 (2)	H16A—C16—H16C	109.5
O1—C8—C9	115.66 (18)	H16B—C16—H16C	109.5
C8—C9—H9A	109.5		
			
O2—S1—C1—C8	128.69 (18)	C1—C2—C7—C6	−179.4 (2)
C10—S1—C1—C8	−120.49 (18)	C2—C1—C8—O1	−0.3 (2)
O2—S1—C1—C2	−50.8 (2)	S1—C1—C8—O1	−179.90 (14)
C10—S1—C1—C2	60.1 (2)	C2—C1—C8—C9	−179.9 (2)
C8—C1—C2—C3	−179.0 (2)	S1—C1—C8—C9	0.5 (4)
S1—C1—C2—C3	0.5 (4)	C7—O1—C8—C1	0.3 (2)
C8—C1—C2—C7	0.2 (2)	C7—O1—C8—C9	−179.99 (18)
S1—C1—C2—C7	179.73 (17)	O2—S1—C10—C15	−3.5 (2)
C7—C2—C3—C4	1.6 (3)	C1—S1—C10—C15	−116.2 (2)
C1—C2—C3—C4	−179.3 (2)	O2—S1—C10—C11	−178.65 (17)
C2—C3—C4—C5	−1.5 (3)	C1—S1—C10—C11	68.59 (19)
C2—C3—C4—Br1	177.05 (15)	C15—C10—C11—C12	1.5 (3)
C3—C4—C5—C6	−0.2 (4)	S1—C10—C11—C12	176.65 (18)
Br1—C4—C5—C6	−178.77 (17)	C10—C11—C12—C13	−1.1 (4)
C4—C5—C6—C7	1.7 (3)	C11—C12—C13—C14	−0.1 (4)
C8—O1—C7—C6	179.2 (2)	C11—C12—C13—C16	179.9 (2)
C8—O1—C7—C2	−0.2 (2)	C12—C13—C14—C15	1.0 (4)
C5—C6—C7—O1	179.0 (2)	C16—C13—C14—C15	−179.0 (3)
C5—C6—C7—C2	−1.7 (3)	C11—C10—C15—C14	−0.6 (4)
C3—C2—C7—O1	179.38 (18)	S1—C10—C15—C14	−175.7 (2)
C1—C2—C7—O1	0.0 (2)	C13—C14—C15—C10	−0.7 (4)
C3—C2—C7—C6	0.0 (3)		

Symmetry code: (i) *x*, −*y*+1/2, *z*+1/2.

### Hydrogen-bond geometry (Å, º)

**Table:**

*D*—H···*A*	*D*—H	H···*A*	*D*···*A*	*D*—H···*A*
C5—H5···O1^ii^	0.95	2.49	3.420 (3)	165
C9—H9*C*···O2^iii^	0.98	2.48	3.294 (3)	141

Symmetry codes: (ii) −*x*+2, *y*−1/2, −*z*+3/2; (iii) *x*, −*y*+3/2, *z*+1/2.
